# Septicemia and mortality after noncardiac surgery associated with CHA2DS2-VASc score: a retrospective cohort study based on a real-world database

**DOI:** 10.1186/s12893-021-01209-z

**Published:** 2021-04-26

**Authors:** Li-Chin Sung, Chih-Chung Liu, Chao-Shun Lin, Chun-Chieh Yeh, Yih-Giun Cherng, Ta-Liang Chen, Chien-Chang Liao

**Affiliations:** 1 Division of Cardiology, Department of Internal Medicine, School of Medicine, College of Medicine, Taipei Medical University, Taipei City, Taiwan; 2 Division of Cardiology, Department of Internal Medicine, Shuang Ho Hospital, Taipei Medical University, New Taipei City, Taiwan; 3 Taipei Heart Institute, Taipei Medical University, Taipei, Taiwan; 4 Department of Anesthesiology, Taipei Medical University Hospital, Taipei, Taiwan; 5 Anesthesiology and Health Policy Research Center, Taipei Medical University Hospital, Taipei, Taiwan; 6 Department of Anesthesiology, School of Medicine, College of Medicine, Taipei Medical University, Taipei, Taiwan; 7 Department of Surgery, China Medical University Hospital, Taichung, Taiwan; 8 Department of Surgery, University of Illinois, Chicago, USA; 9 Department of Anesthesiology, Shuang Ho Hospital, Taipei Medical University, New Taipei City, Taiwan; 10 Department of Anesthesiology, Wan Fang Hospital, Taipei Medical University, Taipei, Taiwan; 11 Research Center of Big Data and Meta-Analysis, Wan Fang Hospital, Taipei Medical University, Taipei, Taiwan; 12 School of Chinese Medicine, College of Chinese Medicine, China Medical University, Taichung, Taiwan

**Keywords:** Atrial fibrillation, CHA2DS2-VASc score, Infectious complications, Mortality, Surgery

## Abstract

**Background:**

Little was know about the association between the CHA2DS2-VASc score and postoperative outcomes. Our purpose is to evaluate the effects of CHA2DS2-VASc score on the perioperative outcomes in patients with atrial fibrillation (AF).

**Methods:**

We identified 47,402 patients with AF over the age of 20 years who underwent noncardiac surgeries between 2008 and 2013 from claims data of the National Health Insurance in Taiwan. The CHA2DS2-VASc score was used to evaluate postoperative complications, mortality and the consumption of medical resources by calculating adjusted odds ratios (ORs) and 95% confidence intervals (CIs).

**Results:**

Compared with patients with a CHA2DS2-VASc score of 0, patients with scores ≥ 5 had an increased risk of postoperative septicemia (OR 2.76, 95% CI 2.00–3.80), intensive care (OR 2.55, 95% CI 2.12–3.06), and mortality (OR 2.04, 95% CI 1.14–3.64). There was a significant positive correlation between risk of postoperative complication and the CHA2DS2-VASc score (P < 0.0001).

**Conclusion:**

The CHA2DS2-VASc score was highly associated with postoperative septicemia, intensive care, and 30-day mortality among AF patients. Cardiologists and surgical care teams may consider using the CHA2DS2-VASc score to evaluate perioperative outcome risks in patients with AF.

**Supplementary Information:**

The online version contains supplementary material available at 10.1186/s12893-021-01209-z.

## Background

Atrial fibrillation (AF) is the most common sustained cardiac arrhythmia with an estimated five million incident cases worldwide [1, 2]. The 2010 global burden of disease study reported that there were 33.5 million patients with AF globally, constituting approximately 0.5% of the total world population [2]. The incidence of AF increases dramatically with age and is higher in men than women [3]. Numerous studies have reported a lifetime risk of developing AF among those aged ≥ 40 years of approximately 20–25% [3]. Furthermore, this prevalence is likely underestimated since a large number of asymptomatic individuals and those having transient symptoms remain undiagnosed. AF is associated with an increased risk of thromboembolic stroke, acute coronary syndrome, heart failure, chronic kidney disease, hospitalization and all-cause mortality, as well as higher medical costs and a reduced quality of life [1, 4–6].

The CHA2DS2-VASc score for stroke risk assessment in patients with AF is well validated and has been widely applied and adopted in the U.S. and European clinical guidelines, as well as in the Asia Pacific Heart Rhythm Society, as a basic risk assessment tool [7–11]. The CHA2DS2-VASc score may predict adverse cardiovascular events and mortality in subsets of certain populations with high accuracy [12–14]. Moreover, the CHA2DS2-VASc score has also been shown to be useful in predicting ischemic stroke even among individuals without AF [12, 15].

Since the number of people with AF is increasing, more AF patients will require risk stratification before surgery [1, 2]. Clinical guidelines for perioperative risk assessment focus on coronary artery disease rather than AF as an important risk factor for adverse outcomes [16, 17]. In a population-based data analysis of 38,047 consecutive patients, the 30-day postoperative mortality rate was significantly higher in patients with AF than in those with coronary heart disease [18]. Infectious complications are the major causes of postoperative morbidity and mortality in noncardiac surgery, which merits increased attention and intervention [19]. Some studies have also reported that AF is strongly associated with hospital-acquired pneumonia or postoperative infection [20]. However, limited information is available regarding the potential application of the CHA2DS2-VASc score to adverse outcomes in AF patients receiving noncardiac surgeries. Thus, we used reimbursement claims from the Taiwan Health Insurance Research Database to conduct a population-based cohort study to investigate whether the CHA2DS2-VASc score is associated with the relative risk of postoperative adverse events in patients with AF when receiving noncardiac surgeries.

## Methods

### Source of data

In this study, we used the Taiwan Health Insurance Research Database from the National Health Insurance in Taiwan, which was has been in place since 1995 and covers more than 99% of all residents. Basic patient characteristics, physician diagnoses, treatment procedures, medications, and consumption of medical resources were recorded in the database. Detailed information described in previous reports and articles based on this database was assessed scientifically and published in important journals [21, 22].

The data underlying this study is from the Health and Welfare Data Science Center, Department of Statistics, Ministry of Health and Welfare, Taiwan. Interested researchers can obtain the data through formal application to the Health and Welfare Data Science Center (http://dep.mohw.gov.tw/DOS/np-2497-113.html) and contact the agency with email. We have made the formal application (included application documents, study proposals, and ethics approval of the institutional review board) of the current insurance data. The authors of the present study had no special access privileges in accessing the data which other interested researchers would not have.

Under the regulations from the National Health Insurance In accordance with the Helsinki Declaration and in order to protect personal privacy, patient identifications were scrambled and decoded. Our study was evaluated and approved by the Institutional Review Board of Taipei Medical University (TMU-JIRB-201905042; TMU-JIRB-201902053; TMU-JIRB-201808012; TMU-JIRB-201710033; TMU-JIRB-201701050; TMU-JIRB-201506001) and was exempted from the requirement for informed patient consent.

### Study design

In the research database of the National Health Insurance, we identified 3,639,792 patients aged ≥ 20 years who underwent major surgeries (requiring general anesthesia or neuraxial anesthesia with hospitalization for more than one day) in 2008–2013. Of these, 47,402 had a history of AF assessed by the CHA2DS2-VASc score and categorized with scores of 0 and ≥ 5. We analyzed postoperative infection-related complications, mortality, and consumption of medical resources among AF patients with various CHA2DS2-VASc scores.

### Criteria and definition

The components of the CHA2DS2-VASc score included the following: age ≥ 75 years (2 points), stroke/transient ischemic attack/thromboembolic event (2 points), age 65–74 years (1 point), female sex (1 point), congestive heart failure (1 point), hypertension (1 point), diabetes (1 point), and vascular disease (1 point). Low income is defined as individuals who utilize medical care services without paying the Taiwanese National Health Insurance copayment.

We used *the International Classification of Diseases, Ninth Revision, Clinical Modification* (ICD-9-CM) and administration codes to identify physician diagnoses of disease histories and complications after surgery in the Taiwan Health Insurance Research Database. Based on our previous studies, we included surgical patients’ current medical conditions and their history of diseases such as mental disorders, chronic obstructive pulmonary disease, cancer, chronic kidney disease, hyperlipidemia, renal dialysis, Parkinson’s disease, and liver cirrhosis as covariates in this study. Postoperative infection-related complications, such as pneumonia, septicemia, and urinary tract infection, were also identified. During the index surgical hospitalization, the consumption of medical resources including admission to an intensive care unit, length of hospital stay, and medical expenditure were also considered as study outcomes. In this study, we defined the postoperative adverse events as surgical patients has any of one complications or mortality during the index surgical hospitalization included postoperative pneumonia, septicemia, urinary tract infection, and 30-day in-hospital mortality.

### Statistical analysis

We used chi-square tests to compare categorical variables (summarized using frequency and percentage) between AF patients with a CHA2DS2-VASc score of 0 and ≥ 1. Continuous variables were compared using a *t*-test (summarized using mean ± SD). We used multivariate logistic regression to calculate the odds ratios (ORs) and 95% confidence intervals (CIs) of the CHA2DS2-VASc score associated with postoperative pneumonia, septicemia, urinary tract infection, intensive care unit stay, and in-hospital mortality. Multiple linear regressions were used to evaluate the relationship between the CHA2DS2-VASc score and length of hospital stay and medical expenditure. Adjusted ORs (95% CIs) of postoperative adverse events for patients with each component of the CHA2DS2-VASc score were also calculated. Multiple logistic regressions were also used to calculate adjusted ORs (95% CIs) of the CHA2DS2-VASc score associated with postoperative adverse events in the subgroups of male gender, number of medical conditions and types of anesthesia.

## Results

Among 47,402 surgical patients with AF (Additional file 1: Table S1), 45,639 (96.3%) had a CHA2DS2-VASc score of ≥ 1. Compared with AF patients with a CHA2DS2-VASc score of 0 (Table 1), those with score of ≥ 1 had higher incidences of mental disorders (*P* < 0.0001), liver cirrhosis (*P* < 0.0001), chronic obstructive pulmonary disease (*P* < 0.0001), stroke (*P* < 0.0001), chronic kidney disease (*P* = 0.003), and Parkinson’s disease (*P* < 0.0001). The incidence of low income was higher in AF patients with scores of 0 compared with AF patients with scores of ≥ 1 (*P* = 0.0004).

**Table 1:** Characteristics of surgical patients with atrial fibrillation by CHA2DS2-VASc score

	0 score (N = 1763)		≥ 1 score (N = 45,639)		p-value
	n	(%)	n	(%)	
Sex					
Female	0	(0.0)	19,644	(43.0)	< 0.0001
Male	1763	(100.0)	25,995	(57.0)	
Age, years					
20–34	69	(3.9)	148	(0.3)	< 0.0001
35–44	158	(9.0)	438	(1.0)	
45–54	482	(27.3)	1834	(4.0)	
55–64	1054	(59.8)	5678	(12.4)	
65–74	0	(0.0)	12,622	(27.7)	
≥ 75	0	(0.0)	24,919	(54.6)	
Low income					
No	1709	(96.9)	44,402	(97.3)	0.3722
Yes	54	(3.1)	1237	(2.7)	
Medical conditions					
Mental disorders	313	(17.8)	10,719	(23.5)	< 0.0001
COPD	110	(6.2)	8887	(19.5)	< 0.0001
Cancer	280	(15.9)	7648	(16.8)	0.3337
Stroke	0	(0.0)	6865	(15.0)	< 0.0001
CKD	79	(4.5)	3973	(8.7)	< 0.0001
Hyperlipidemia	70	(4.0)	2206	(4.8)	0.0963
Renal dialysis	62	(3.5)	2200	(4.8)	0.0117
Parkinson’s disease	7	(0.4)	1877	(4.1)	< 0.0001
Liver cirrhosis	85	(4.8)	1359	(3.0)	< 0.0001
Obesity	10	(0.6)	312	(0.7)	0.5593
Types of surgery					
Skin	41	(2.3)	1063	(2.3)	< 0.0001
Breast	1	(0.1)	333	(0.7)	
Musculoskeletal	402	(22.8)	15,119	(33.1)	
Respiratory	144	(8.2)	1988	(4.4)	
Digestive	607	(34.4)	12,173	(26.7)	
Kidney, ureter, bladder	169	(9.6)	4216	(9.2)	
Delivery, CS, abortion	0	(0.0)	59	(0.1)	
Neurosurgery	221	(12.5)	6068	(13.3)	
Eye	20	(1.1)	441	(1.0)	
Others	158	(9.0)	4179	(9.2)	
Types of anesthesia					
General	1360	(77.1)	31,836	(69.8)	< 0.0001
Epidural or Spinal	403	(22.9)	13,803	(30.2)	

*AF* atrial fibrillation

Postoperative infection-related complications, such as pneumonia (*P* < 0.0001), septicemia (*P* < 0.0001), and urinary tract infection (*P* < 0.0001) were associated with the CHA2DS2-VASc score with a biological gradient trend. Compared to AF patients with a CHA2DS2-VASc score of 0 (Table 2), those with scores of ≥ 5 showed an increased risk of postoperative pneumonia septicemia (OR = 2.76; 95% CI 2.00–3.80), intensive care (OR = 2.55; 95% CI 2.12–3.06) and in-hospital mortality (OR = 2.04; 95% CI 1.14–3.64) with significant trends (*P* for trend were < 0.0001 for both). In Table 3, CHA2DS2-VASc score ≥ 5 was associated with postoperative adverse events in patients received musculoskeletal surgery (OR = 4.40; 95% CI 2.30–7.09) and digestive surgery (OR = 1.67; 95% CI 1.14–2.45).

**Table 2:** Adverse outcomes after non-cardiac surgeries associated with CHA2DS2-VASc score in patients with atrial fibrillation

Postoperative outcomes	Scores	N	Risk of outcome		
			Events	Incidence,%	OR (95% CI)^†^
Pneumonia	0	1763	56	3.2	1.00 (reference)
	1	7214	408	5.7	1.22 (0.90–1.66)
	2	12,419	857	6.9	1.24 (0.91–1.69)
	3	12,894	889	6.9	1.15 (0.84–1.58)
	4	8314	699	8.4	1.30 (0.94–1.79)
	≥ 5	4798	435	9.1	1.31 (0.94–1.83)
Septicemia	0	1763	55	3.1	1.00 (reference)
	1	7214	471	6.5	1.92 (1.43–2.58)
	2	12,419	950	7.7	2.07 (1.54–2.79)
	3	12,894	1071	8.3	2.19 (1.62–2.95)
	4	8314	836	10.1	2.53 (1.86–3.44)
	≥ 5	4798	556	11.6	2.76 (2.00–3.80)
Urinary tract infection	0	1763	57	3.2	1.00 (reference)
	1	7214	500	6.9	1.29 (0.96–1.73)
	2	12,419	991	8.0	1.22 (0.90–1.64)
	3	12,894	1197	9.3	1.30 (0.96–1.76)
	4	8314	825	9.9	1.29 (0.94–1.76)
	≥ 5	4798	523	10.9	1.36 (0.98–1.88)
Infectious complications*	0	1763	148	8.4	1.00 (reference)
	1	7214	1160	16.1	1.50 (1.24–1.82)
	2	12,419	2340	18.8	1.56 (1.29–1.89)
	3	12,894	2612	20.3	1.61 (1.32–1.96)
	4	8314	1892	22.8	1.73 (1.42–2.12)
	≥ 5	4798	1237	25.8	1.93 (1.56–2.38)
Intensive care	0	1763	254	14.4	1.00 (reference)
	1	7214	1485	20.6	1.38 (1.18–1.61)
	2	12,419	3047	24.5	1.55 (1.32–1.82)
	3	12,894	3412	26.5	1.67 (1.42–1.97)
	4	8314	2659	32.0	2.15 (1.81–2.55)
	≥ 5	4798	1715	35.7	2.55 (2.12–3.06)
30-day in-hospital mortality	0	1763	18	1.0	1.00 (reference)
	1	7214	88	1.2	1.08 (0.64–1.85)
	2	12,419	241	1.9	1.57 (0.93–2.66)
	3	12,894	246	1.9	1.55 (0.90–2.65)
	4	8314	195	2.4	1.87 (1.08–3.25)
	≥ 5	4798	121	2.5	2.04 (1.14–3.64)

*CI* confidence interval, *OR* odds ratio

*Infectious complications included with pneumonia, septicemia, and urinary tract infection

^†^Adjusted for all covariates listed in Table 1

**Table 3:** Stratified analysis for risk of postoperative adverse events associated with CHA2DS2-VASc score in patients with atrial fibrillation

	Scores	N	Postoperative adverse events*		
			Events	Incidence,%	OR (95% CI)^†^
Musculoskeletal surgery	0	402	16	4.0	1.00 (reference)
	1	1662	201	12.1	2.43 (1.41–4.19)
	2	3550	500	14.1	2.57 (1.49–4.43)
	3	4527	742	16.4	3.07 (1.78–5.32)
	4	3286	613	18.7	3.42 (1.96–5.94)
	≥ 5	2094	464	22.2	4.04 (2.30–7.09)
Digestive surgery	0	607	47	7.7	1.00 (reference)
	1	2259	342	15.1	1.39 (0.99–1.95)
	2	3633	729	20.1	1.58 (1.12–2.22)
	3	3366	754	22.4	1.51 (1.07–2.14)
	4	1887	492	26.1	1.54 (1.07–2.21)
	≥ 5	1028	315	30.6	1.67 (1.14–2.45)
Neurosurgery	0	221	41	18.6	1.00 (reference)
	1	843	165	19.6	0.87 (0.59–1.29)
	2	1630	389	23.9	0.98 (0.66–1.46)
	3	1773	429	24.2	0.94 (0.63–1.42)
	4	1189	301	25.3	0.92 (0.60–1.42)
	≥ 5	633	184	29.1	1.05 (0.66–1.67)
Respiratory surgery	0	144	15	10.4	1.00 (reference)
	1	399	104	26.1	1.54 (0.82–2.88)
	2	546	182	33.3	1.52 (0.80–2.87)
	3	480	191	39.8	1.65 (0.85–3.19)
	4	348	157	45.1	1.94 (0.98–3.85)
	≥ 5	215	104	48.4	1.82 (0.86–3.81)

*CI* confidence interval, *OR* odds ratio

*Postoperative adverse events included with pneumonia, septicemia, urinary tract infection, and 30-day in-hospital mortality

^†^Adjusted for all covariates listed in Table 1

Compared to individuals with a CHA2DS2-VASc score of 0 (Table 4), patients aged ≥ 75 years (OR = 3.53; 95% CI 2.98–4.18) had the highest risk of postoperative adverse events followed by those with stroke (OR = 2.14; 95% CI 1.78–2.58), an age of 65–74 years (OR = 2.13; 95% CI 1.79–2.52), congestive heart failure (OR = 1.93; 95% CI 1.60–2.31), vascular disease (OR = 1.51; 95% CI 1.26–1.81), diabetes (OR = 1.82; 95% CI 1.51–2.18), female gender (OR = 1.62; 95% CI 1.35–1.94), and hypertension (OR = 1.53; 95% CI 1.28–1.84). The adjusted ORs of postoperative adverse events associated with CHA2DS2-VASc score of 1, 2, 3, 4, ≥ 5 were 1.47 (95% CI 1.22–1.76), 1.55, (95% CI 1.29–1.87), 1.62 (95% CI 1.33–1.95), 1.74 (95% CI 1.44–2.12), and 1.96 (95% CI 1.60–2.41), respectively.

**Table 4:** Adverse events after non-cardiac surgeries associated with components of CHA2DS2-VASc score

Components of CHA2DS2-VASc score	N	Postoperative adverse events*		
		Events	Rate, %	OR (95% CI)^†^
Control group (0 score)	1763	160	9.1	1.00 (reference)
Patient with				
Age ≥ 75 years (≥ 2 scores)	24,919	6394	25.7	3.53 (2.98–4.18)
Stroke (≥ 2 scores)	6865	1949	28.4	2.14 (1.78–2.58)
Age 65–74 years (≥ 1 score)	12,622	2215	17.6	2.13 (1.79–2.52)
Congestive heart failure (≥ 1 score)	11,232	2934	26.1	1.93 (1.60–2.31)
Vascular disease (≥ 1 score)	13,488	2871	21.3	1.51 (1.26–1.81)
Diabetes (≥ 1 score)	12,491	2930	23.5	1.82 (1.51–2.18)
Female (≥ 1 score)	19,644	3979	20.3	1.62 (1.35–1.94)
Hypertension (≥ 1 score)	27,894	5972	21.4	1.53 (1.28–1.84)
CHA2DS2-VASc score				
0	1763	160	9.1	1.00 (reference)
1	7214	1205	16.7	1.47 (1.22–1.76)
2	12,419	2458	19.8	1.55 (1.29–1.87)
3	12,894	2750	21.3	1.61 (1.33–1.95)
4	8314	1994	24.0	1.74 (1.44–2.12)
≥ 5	4798	1310	27.3	1.96 (1.60–2.41)

*CI* confidence interval, *OR* odds ratio

*Postoperative adverse events included with pneumonia, septicemia, urinary tract infection, and mortality

^†^Adjusted for all covariates listed in Table 1

In Additional file 1: Table S2, the average hospital stay length (*P* < 0.0001) and medical expenditure (*P* < 0.0001) were higher in surgical patients with a CHA2DS2-VASc score of ≥ 5 compared to surgical patients with a CHA2DS2-VASc score of 0. After adjusting for covariates in the multiple regression analysis, the CHA2DS2-VASc score was significantly associated with the length of hospital stay (beta = 1.25, *P* < 0.0001) and medical expenditure (beta = 256, *P* < 0.0001).

Additional file 1: Table S3 shows a stratified analysis of the risk of postoperative adverse events in association with a CHA2DS2-VASc score in patients with AF. Among patients with AF, a CHA2DS2-VASc score of ≥ 4 is a significant risk factor for adverse events after noncardiac surgeries in the following subgroups: male (OR = 2.11; 95% CI 1.72–2.58), patients with 0 medical conditions (OR = 2.49; 95% CI 1.88–3.28), patients with 1 medical condition (OR = 1.79; 95% CI 1.31–2.45), patients with ≥ 2 medical conditions (OR = 2.50; 95% CI 1.53–4.09), epidural/spinal anesthesia (OR = 2.43; 95% CI 1.50–3.93), and general anesthesia (OR = 2.06; 95% CI 1.67–2.54).

## Discussion

This study is the first population-based study to report the application of the CHA2DS2-VASc score to assessments of perioperative of noncardiac surgery outcomes in patients with AF. The CHA2DS2-VASc score was highly associated with postoperative major infection, intensive care unit stay, and 30-day mortality. Prolonged length of hospital stay and elevated medical expenditures were also noted in patients with higher CHA2DS2-VASc scores. The biological gradient effects existed in the CHA2DS2-VASc score associated with postoperative infections and mortality. The association between CHA2DS2-VASc score and postoperative adverse events remained significant for various subgroups.

Age, sex, and socioeconomic covariates commonly influence perioperative outcomes [23–25]. Postoperative complications are mainly determined by types of surgery and anesthesia, as well as pre-existing medical conditions such as hyperlipidemia, mental disorder, liver cirrhosis, renal disease, chronic obstructive pulmonary disease, Parkinson’s disease and cancer. These conditions are also considered to be potential associated factors of postoperative complications and mortality [23, 26–31]. To avoid bias when investigating the relationships between the CHA2DS2-VASc score and postoperative outcomes, we used multivariate logistic regression models to adjust for these potential confounding factors. We then showed that the CHA2DS2-VASc score was a statistically significant predictor for postoperative infection and in-hospital mortality in patient with AF.

Recent studies have reported that preoperative AF clearly increases the risk of perioperative stroke and adverse cardiovascular events, as well as short-term and long-term mortality [18, 32, 33]. Whether various CHA2DS2-VASc scores are correlated with other postoperative complications in AF patients undergoing noncardiac surgeries remains unclear. According to the present data, incremental increases in CHA2DS2-VASc scores caused a two- to three-fold risk in infection-related postoperative complications. The possible explanation is that each component of the scoring systems was proven to be independently associated with higher risks of postoperative infection and mortality [20, 21, 25, 34–36]. In our previous report and present investigation, patients with a previous stroke had double the risk of postoperative mortality than patients without previous stroke, either in the entire or AF populations [21]. Moreover, each of these factors has been independently recognized to affect outcomes in AF. The CHA2DS2-VASc score predicts stroke risk in AF patients and research has shown that up to 50% of patients who had acute stroke have clinical evidence of aspiration pneumonia or sepsis, demonstrating its predictive role for infectious outcomes [20, 37]. On the basis of these findings, we postulate that the CHA2DS2-VASc score is strongly associated with postoperative complications in AF patients. However, this assumption should be validated in future prospective randomized trials.

Preoperative AF was independently associated with higher postoperative complications in patients undergoing noncardiac surgery [18]. In the current clinical settings, the Revised Cardiac Risk Index or the American College of Surgeons National Surgical Quality Improvement Program risk model calculator were used to assess the cardiovascular risk in all patients who were scheduled to undergo noncardiac surgeries [38]. According to the clinical practice guidelines, electrocardiogram and echocardiography are common tools for assessing perioperative outcomes that are not recognized as risks in aforementioned risk model calculators [16, 17, 38]. However, the comprehensive assessment of preoperative risk stratification in AF population has not been ideally established. Therefore, our study examined the possibility of applying the CHA2DS2-VASc score to surgical patients in order to assess infectious complications and mortality.

A retrospective population-based cohort study was conducted to investigate which risk score for perioperative outcomes prediction in patients with AF undergoing noncardiac surgery and concluded that the CHA2DS2-VASc score provides acceptable preoperative risk stratification for major perioperative events including mortality [26]. Compared with our study, the authors did not adjust some possible potential confounding factors into their analysis (such as socioeconomic status, hyperlipidemia, liver cirrhosis and types of anesthesia). Additionally, the patient cohort in our study was larger and from a more recent time period compared with the previous cohort study [26]. Furthermore, the present study provides evidence that patients with AF with an increased CHA2DS2-VASc score have an increased risk for postoperative adverse events compared to patients with AF only. With these results, clinicians can precisely access the risk of adverse outcomes and allocate medical resources in AF patients with coexisting medical conditions when they undergo noncardiac surgeries.

This study had several inherent limitations. First, several unmeasured factors, such as the type of AF (paroxysmal or non-paroxysmal), frailty, various perioperative AF medication management strategies, drug compliance, alcohol consumption, body mass index, cigarette smoking, physical activity level, and perioperative heart rate status, were unavailable in our database. Failure to consider the aforementioned variables may have led to a certain degree of residual bias. However, considering the significance and magnitude of the observed effects, it is unlikely that these limitations compromised the results. Second, comorbidity severities (such as CHA2DS2-VASc score components) and coexisting medical conditions were defined by registered diagnosis codes, not by laboratory data, image studies or clinical evaluations. In addition, because the study cohort included only Taiwanese patients with AF, the results may not be generalizable to other populations. Finally, our investigation was a retrospective observational study, which had certain methodological limitations. Understanding the causal inference between the CHA2DS2-VASc score and perioperative outcomes requires future prospective studies.

## Conclusions

In conclusion, the CHA2DS2-VASc scoring system is an important independent predictor for postoperative septicemia, 30-day mortality, and consumption of medical resources in patients with AF undergoing noncardiac surgeries. Our study suggests that perioperative care teams could apply CHA2DS2-VASc scores preoperatively for AF patients receiving noncardiac surgeries. Future studies are needed to assess the application of CHA2DS2-VASc scores to AF patients undergoing noncardiac surgeries.

## Supplementary Information

**Additional file 1: Table S1.** Characteristics of surgical patients with atrial fibrillation. **Table S2.** Length of hospital stay and medical expenditures associated with CHA2DS2-VASc score in patients with atrial fibrillation. **Table S3.** Stratified analysis for risk of postoperative adverse events associated with CHA2DS2-VASc score in patients with atrial fibrillation.

## Abbreviations

AF: Atrial fibrillation
ICD-9-CM: International code of diseases, ninth edition, clinical modification
OR: Odds ratio
CI: Confidence interval
