# Supplementary material for: Septicemia and mortality after noncardiac surgery associated with CHA2DS2-VASc score: a retrospective cohort study based on a real-world database
Source: BMC Surg. 2021 Apr 26;21:209. doi: 10.1186/s12893-021-01209-z (PMC8073955; doi:10.1186/s12893-021-01209-z)
Supplement: Supplementary file 1 — Additional file 1: Table S1. Characteristics of surgical patients with atrial fibrillation. Table S2. Length of hospital stay and medical expenditures associated with CHA2DS2-VASc score in patients with atrial fibrillation. Table S3. Stratified analysis for risk of postoperative adverse events associated with CHA2DS2-VASc score in patients with atrial fibrillation. [file 12893_2021_1209_MOESM1_ESM.doc]

| **Table S1** Characteristics of surgical patients with atrial fibrillation | | |
| --- | --- | --- |
|  | Total (N=47402) | |
|  | n | (%) |
| CHA2DS2-VASc scores |  |  |
| 0 | 1763 | (3.7) |
| 1 | 7214 | (15.2) |
| 2 | 12419 | (26.2) |
| 3 | 12894 | (27.2) |
| 4 | 8314 | (17.5) |
| 5 | 3717 | (7.8) |
| 6 | 987 | (2.1) |
| 7 | 94 | (0.2) |
| Sex |  |  |
| Female | 19644 | (41.4) |
| Male | 27758 | (58.6) |
| Age, years |  |  |
| 20-34 | 217 | (0.5) |
| 35-44 | 596 | (1.3) |
| 45-54 | 2316 | (4.9) |
| 55-64 | 6732 | (14.2) |
| 65-74 | 12622 | (26.6) |
| ≥75 | 24919 | (52.6) |
| Low income |  |  |
| No | 46111 | (97.3) |
| Yes | 1291 | (2.7) |
| Medical conditions |  |  |
| Hyperlipidemia | 2276 | (4.8) |
| Mental disorders | 11032 | (23.3) |
| Liver cirrhosis | 1444 | (3.1) |
| Chronic kidney disease | 4052 | (8.6) |
| Renal dialysis | 2262 | (4.8) |
| COPD | 8997 | (19.0) |
| Parkinson’s disease | 1884 | (4.0) |
| Cancer | 7928 | (16.7) |
| Types of surgery |  |  |
| Skin | 1104 | (2.3) |
| Breast | 334 | (0.7) |
| Musculoskeletal | 15521 | (32.7) |
| Respiratory | 2132 | (4.5) |
| Digestive | 12780 | (27.0) |
| Kidney, ureter, bladder | 4385 | (9.3) |
| Delivery, CS, abortion | 59 | (0.1) |
| Neurosurgery | 6289 | (13.3) |
| Eye | 461 | (1.0) |
| Others | 4337 | (9.2) |
| Types of anesthesia |  |  |
| General | 33196 | (70.0) |
| Epidural or Spinal | 14206 | (30.0) |
| AF, Atrial fibrillation; COPD, chronic obstructive pulmonary disease. | | |

| **Table S2** Length of hospital stay and medical expenditures associated with CHA2DS2-VASc score in patients with atrial fibrillation* | | | | | | | |
| --- | --- | --- | --- | --- | --- | --- | --- |
| CHA2DS2-VASc |  | Medical expenditure, USD | | | Length of hospital stay, days | | |
| Score | n | Mean | ± | SD | Mean | ± | SD |
| 0 | 1763 | 3096 | ± | 4404 | 8.7 | ± | 11.6 |
| 1 | 7214 | 3632 | ± | 5161 | 10.5 | ± | 13.2 |
| 2 | 12419 | 4025 | ± | 4982 | 12.0 | ± | 14.9 |
| 3 | 12894 | 4203 | ± | 4913 | 13.0 | ± | 16.3 |
| 4 | 8314 | 4564 | ± | 4928 | 14.5 | ± | 17.1 |
| ≥5 | 4798 | 4945 | ± | 5320 | 16.3 | ± | 20.0 |
| p-value |  | < 0.0001 | | | < 0.0001 | | |
| *Multiple regression analysis for medical expenditure (beta=256, 95% CI=216-298, *P* < 0.0001) and length of hospital stay (beta=1.25, 95% CI=1.11-1.38, *P* < 0.0001) associated with CHA2DS2-VASc score. | | | | | | | |

| **Table S3** Stratified analysis for risk of postoperative adverse events associated with CHA2DS2-VASc score in patients with atrial fibrillation | | | | | | |
| --- | --- | --- | --- | --- | --- | --- |
|  |  |  | Postoperative adverse events* | | | |
|  | Scores | N | Events | Incidence,% | OR | (95% CI)† |
| Female | 0 | 0 | 0 | 0.0 | 1.00 | (reference) |
|  | 1 | 1183 | 106 | 9.0 | - | - |
|  | 2 | 3439 | 572 | 16.6 | - | - |
|  | 3 | 6238 | 1215 | 19.5 | - | - |
|  | ≥4 | 8784 | 2086 | 23.8 | - | - |
| Male | 0 | 1763 | 160 | 9.1 | 1.00 | (reference) |
|  | 1 | 6031 | 1099 | 18.2 | 1.57 | (1.30-1.90) |
|  | 2 | 8980 | 1886 | 21.0 | 1.64 | (1.35-1.99) |
|  | 3 | 6656 | 1535 | 23.1 | 1.72 | (1.41-2.09) |
|  | ≥4 | 4328 | 1218 | 28.1 | 2.11 | (1.72-2.58) |
| 0 medical condition | 0 | 1010 | 77 | 7.6 | 1.00 | (reference) |
|  | 1 | 3548 | 499 | 14.1 | 1.61 | (1.24-2.09) |
|  | 2 | 5495 | 953 | 17.3 | 1.84 | (1.41-2.39) |
|  | 3 | 5397 | 1058 | 19.6 | 2.14 | (1.63-2.80) |
|  | ≥4 | 4919 | 1125 | 22.9 | 2.49 | (1.88-3.28) |
| 1 medical condition | 0 | 545 | 62 | 11.4 | 1.00 | (reference) |
|  | 1 | 2520 | 445 | 17.7 | 1.21 | (0.89-1.63) |
|  | 2 | 4525 | 939 | 20.8 | 1.33 | (0.98-1.80) |
|  | 3 | 4721 | 1046 | 22.2 | 1.42 | (1.04-1.93) |
|  | ≥4 | 4779 | 1253 | 26.2 | 1.79 | (1.31-2.45) |
| ≥2 medical conditions | 0 | 208 | 21 | 10.1 | 1.00 | (reference) |
|  | 1 | 1146 | 261 | 22.8 | 2.00 | (1.23-3.26) |
|  | 2 | 2399 | 566 | 23.6 | 1.96 | (1.21-3.18) |
|  | 3 | 2776 | 646 | 23.3 | 1.94 | (1.19-3.15) |
|  | ≥4 | 3414 | 926 | 27.1 | 2.50 | (1.53-4.09) |
| Epidural/spinal anesthesia | 0 | 403 | 23 | 5.7 | 1.00 | (reference) |
|  | 1 | 2114 | 346 | 16.4 | 2.21 | (1.38-3.54) |
|  | 2 | 3842 | 656 | 17.1 | 2.02 | (1.26-3.25) |
|  | 3 | 3976 | 694 | 17.5 | 2.04 | (1.27-3.30) |
|  | ≥4 | 3871 | 796 | 20.6 | 2.43 | (1.50-3.93) |
| General anesthesia | 0 | 1360 | 137 | 10.1 | 1.00 | (reference) |
|  | 1 | 5100 | 859 | 16.8 | 1.39 | (1.13-1.69) |
|  | 2 | 8577 | 1802 | 21.0 | 1.58 | (1.30-1.94) |
|  | 3 | 8918 | 2056 | 23.1 | 1.72 | (1.40-2.11) |
|  | ≥4 | 9241 | 2508 | 27.1 | 2.06 | (1.67-2.54) |
| CI, confidence interval; OR, odds ratio.  *Postoperative adverse events included with pneumonia, septicemia, urinary tract infection, and mortality.  †Adjusted for all covariates listed in Table I. | | | | | | |
